# Latent viruses in older adults: Associations with inflammation and implications for health

**DOI:** 10.1093/geroni/igaf122.4149

**Published:** 2025-12-31

**Authors:** Tanvi Tomar, Brett Messman, Jennifer Graham-Engeland, Erik Knight, Christopher Engeland

**Affiliations:** The Pennsylvania State University, University Park, Pennsylvania, United States; The Pennsylvania State University, University Park, Pennsylvania, United States; The Pennsylvania State University, University Park, Pennsylvania, United States; University of Colorado Boulder, Boulder, Colorado, United States; The Pennsylvania State University, University Park, Pennsylvania, United States

## Abstract

Latent viruses, such as Epstein-Barr Virus (EBV), Cytomegalovirus (CMV), and Human Herpesvirus-6 (HHV-6), are persistent viral infections that can reactivate over time and are highly prevalent in older adults (60+), with prevalence rates ranging from 60% to > 95%. The pathogenic burden exerted over the lifespan by such persistent viral infections is increasingly recognized as a risk factor for poorer immunity and health outcomes in older adults. In this context, the current study explored associations between 1) EBV, CMV, and HHV-6 antibody seropositivity or 2) pathogenic burden (i.e., number of seropositive viruses) with C-reactive protein (CRP) and basal and lipopolysaccharide (LPS)-stimulated inflammatory cytokines [interleukin-6 (IL-6), interleukin-8 (IL-8), interleukin-10 (IL-10), tumor necrosis factor-alpha (TNF-a), interferon-gamma (IFN-γ)], assessing basal and reactive immune measures, respectively. Participants were healthy older adults (N = 31; ages 60-81; 68% female; 94% White) recruited from central Pennsylvania. All participants were IgG seropositive for at least one latent virus (19% for 1 virus; 58% for 2 viruses; 22% for 3 viruses). Greater pathogenic burden was negatively correlated with LPS-stimulated cytokines: IL-6 (r=-.09, p=.004); IL-8 (r=-.12, p=.038); IL-10 (r=-.04, p=.015); IFN-γ (r=-.09, p=.044); TNF-α (r=-.12, p=.013). Additionally, among individuals with high IgG levels (4th quantile) the strength of the negative correlations was considerably greater for stimulated IL-6 (r=-.26, p<.001), IL-10 (r=-.16, p<.001), and TNF-α (r=-.27, p<.001). These preliminary findings suggest that greater pathogenic burden of latent viruses may impair the ability to mount an appropriate inflammatory response. These results warrant further investigation between cumulative latent viral burden and inflammation among older adults.

